# The Relationship Between Future Time Perspective and Self-Esteem: A Cross-Cultural Study of Chinese and American College Students

**DOI:** 10.3389/fpsyg.2019.01518

**Published:** 2019-07-03

**Authors:** Houchao Lyu, Gang Du, Kimberly Rios

**Affiliations:** ^1^Faculty of Psychology, Southwest University, Chongqing, China; ^2^Department of Psychology, Ohio University, Athens, OH, United States

**Keywords:** future time perspective, time perspective, self-esteem, cross-cultural differences, undergraduates

## Abstract

The present study explored cross-cultural differences in future time perspective (FTP) and self-esteem and investigated whether the relationship between FTP and self-esteem differs between China and America. The FTP Scale and Rosenberg Self-Esteem Scale were administered to 460 Chinese and 340 American undergraduates. Results showed that American undergraduates scored higher on the future-negative, future-positive, future-confusion, future-perseverant, and future-perspicuity subscales than did Chinese undergraduates; American undergraduates also had higher self-esteem than did Chinese undergraduates. The dimensions of FTP (future-negative, future-positive, future-confusion, and future-perseverant) significantly predicted self-esteem in both the Chinese and American samples. These results broaden our understanding of cross-cultural differences in FTP and self-esteem. Implications and future directions are discussed.

## Introduction

Time perspective is an individual-differences variable that influences behavior in various ways (Zimbardo and Boyd, 1999). In recent years, future time perspective (FTP) has acquired a prominent position within research on the psychology of time. FTP is a schema, or experience and conceptualization, of future time, and is operationalized as an individual’s level of cognitive involvement in future life domains (Nuttin, 1985; Seginer and Lens, 2015). Follow-up studies found that FTP includes not only cognitive but also affective, behavioral, and motivational components (Peetsma, 2000; Lyu and Huang, 2016). Therefore, FTP can be defined as an individual’s cognitive, affective, and behavioral tendencies toward the future that are manifested as relatively stable personality traits (Peetsma, 2000; Lyu and Huang, 2016). As a future-oriented personality trait, FTP embodies individual differences in future expectations and predictions; hence, FTP is an important predictor of the actual behaviors of individuals. Results of a meta-analysis revealed significant relationships between FTP and many outcomes (e.g., achievement, well-being, health behavior, risk behavior, retirement planning) (Kooij et al., 2018). However, are FTP scores consistent across different cultural contexts, and how does FTP relate to the self-concept (e.g., self-esteem)? The present study intends to explore these issues.

### Future Time Perspective

The future involves uncertainty and ambiguity, in that risks and opportunities co-exist. Individuals might perceive the future with hope, but the future might also produce feelings of fear (Morselli, 2013), which could be manifested as approach-avoidance conflict. Given the vast cultural differences in people’s economic and social circumstances (Markus and Kitayama, 1991), will individuals with different cultural backgrounds have similar thoughts regarding their future? Mclnerney (2004) stated that researchers should maintain a cautious attitude toward the generalization of conclusions based on Western cultural backgrounds to non-Western cultures. Future consciousness is one concept that may be heavily influenced by culture. In traditional societies, the past and present are more important than the future, and this effect is more salient in cultures with an agricultural or nomadic-based economy (McInerney et al., 1997). For example, studies have shown that North Americans have a strong future orientation (Spears et al., 2001), whereas the Chinese are predominantly past-oriented (Brislin and Kim, 2003). However, such comparisons shed light on preferences for time orientations (e.g., past vs. future) within a single culture, but provide less information about how preferences differ between two or more cultures (Gao, 2016). For instance, although there is evidence showing that North Americans are relatively more focused on the future than on the past, there is no evidence indicating that North Americans are more concerned about the future than East Asians (Gao, 2016). Therefore, to determine whether the latter is actually the case, it is necessary to directly compare North American and East Asian participants’ future orientations.

Morselli (2013) divided FTP into personal FTP and social FTP. Personal FTP refers to the personal achievements embedded within one’s culture, which are not applicable to other cultures. In contrast, social FTP emphasizes the importance of social co-existence, with a focus on long-term goals and goals that transcend personal achievements, as well as the enhancement of intergroup and interpersonal relationships. Although future orientation has been examined in conjunction with other psychological constructs, there is currently insufficient research directly comparing FTP across cultures.

On the one hand, according to research in cultural psychology, American culture emphasizes individualism, freedom, and thinking about the future from one’s own perspective. However, Chinese culture emphasizes collectivism, relationship orientation, and thinking about the future from the perspective of one’s relationship network (Earley, 1989; Forbes et al., 2009; Jiang et al., 2016). Cultural differences may therefore affect the way that individuals think about the future. On the other hand, aspects of the immediate social environment, such as economic prosperity and recession, also affect the way people think about the future (Liebgold, 2014). In other words, people may think about the future according to their society’s current social and economic conditions, and China and America are at different stages of development. Ecological system theory, as proposed by Bronfenbrenner (1979), emphasizes that individuals are embedded in a series of environmental systems (such as culture, society, and shared beliefs) that affect one another. That is, the system interacts with the individual and influences individual development. Investigating whether FTP scores are consistent among individuals with different cultural backgrounds can enhance understanding of the impact of the socio-cultural environment on future orientation generally and FTP specifically.

### Self-Esteem

According to Baumeister et al. (1996), self-esteem is the evaluative component of the self-concept (i.e., the global evaluation of the self). Self-esteem is an important aspect of an individual’s social and cognitive development (Berndt, 2002). Research has shown that self-esteem varies across cultures, such that individuals in Asian countries tend to report lower levels of self-esteem compared to individuals in North America and Western Europe (Farruggia et al., 2004; Cai et al., 2007; Li et al., 2015). These differences in self-esteem might be influenced by differences in individualism (which is more prevalent in Western cultures) and collectivism (which is more prevalent in non-Western cultures). Under individualism, individuals have a tendency toward the expression of autonomy and believe that they are unique within their surroundings. In contrast, under collectivism, individuals regard themselves as similar to others, emphasize social harmony and dependency, and pursue harmonious interpersonal relationships.

### Future Time Perspective and Self-Esteem

We hypothesized that FTP would predict self-esteem. First, self-esteem has been used as an indicator of validity for the FTP scale in several studies (e.g., Zimbardo and Boyd, 1999; Worrell et al., 2015), and these past studies have revealed a positive relationship between FTP and self-esteem (*r* = 0.19, *p* < 0.001; *r* = 0.13, *p* < 0.05, respectively). Second, theoretically, Zaleski (1996) suggests that FTP is the basis for future anxiety, and that the nature of negative events expected and perceived by individuals determines their level of anxiety about the future. Intense anxiety experience triggers a threat to the self-concept and is directly associated with lower self-esteem (Sowislo and Orth, 2013; Zhang et al., 2016). Third, in our daily lives, positive future orientation can promote the improvement of individuals’ self-esteem. For example, future-oriented students will set future goals according to their personal circumstances. If they strive to achieve future goals, they will evaluate themselves positively and hence exhibit higher self-esteem to the extent that they see themselves as meeting these goals. Finally, time balance (namely, remembering the past, grasping the current and planning the future) is conducive to maintenance of mental health, which, to some extent, helps to improve self-esteem.

Pursuing goals, planning, and forming future expectations are daily activities performed by individuals. These goals and expectations reflect self-worth, which requires high self-esteem (Crocker and Wolfe, 2001). We therefore argue that maintaining positive future thinking and high self-esteem are essential to psychological well-being in many societies. It is also possible that differences in sociocultural environments relate to discrepancies between FTP and self-esteem.

### The Present Study

The aims of the current study are to investigate cultural differences in FTP and self-esteem and to test whether cross-cultural consistency exists in the relationship between FTP and self-esteem. We hypothesize that: (1) individuals from two different cultural backgrounds will show differences on FTP dimensions and self-esteem; and (2) within two cultural backgrounds, all FTP dimensions will be significantly correlated with self-esteem, but with differences in predictive power.

## Materials and Methods

### Participants

A total of 819 undergraduates were recruited, of whom 19 participants provided incomplete information and were removed; thus, the final sample consisted of 800 participants. Four hundred and sixty participants were Chinese undergraduates (180 males, 280 females, ages ranged between 17 and 24, *M*age = 19.27, SD = 1.17), who were recruited from universities in Chongqing, China. Three hundred and forty participants were American undergraduates (108 males, 232 females, ages ranged between 18 and 28, *M*age = 20.09, SD = 3.44), who were recruited from the University of Chicago and Ohio University, United States.

### Instruments

The Chinese version of the FTP scale (Lyu and Huang, 2016) was used, which comprises 28 items. Responses were collected via a five-point scale. To obtain an American English version of the FTP scale, the procedure for forward-backward translation recommended by Brislin (1970) was employed. Two psychology professors translated the 28 Chinese items into English, and two bilingual psychology teachers performed back-translation and comparative modifications. Exploratory structural equation modeling (ESEM) analysis, which takes into account the characteristics of exploratory factor analysis and confirmatory factor analysis (Asparouhov and Muthén, 2009), was performed by using Mplus 7.0 (Muthén and Muthén, 2012). The results showed that the model fit for the Chinese sample was good (χ^2^/*df* = 1.78, RMSEA = 0.04, 90% confidence interval [0.03, 0.05], CFI = 0.95, TLI = 0.91, SRMR = 0.03). The American model fit index was also good (χ^2^/*df* = 1.83, RMSEA = 0.07, 90% confidence interval [0.06, 0.08], CFI = 0.90, TLI = 0.86, SRMR = 0.05). In this study, Cronbach’s αs for the subscales ranged from 0.66 to 0.87 among Chinese participants and from 0.65 to 0.80 among American participants (see Table 1).

**Table 1:** Descriptive statistics and ANOVAs.

	China			America			*F* (1, 798)	η^2^
	*M*	SD	α	*M*	SD	α		
Future-negative	**2.12**	0.64	0.87	**2.54**	0.84	0.86	35.94	0.08
Future-positive	**3.56**	0.61	0.84	**3.76**	0.78	0.85	7.67	0.02
Future-confusion	**2.68**	0.70	0.80	**3.00**	0.90	0.78	20.14	0.04
Future-perseverant	**3.58**	0.46	0.70	**3.84**	0.56	0.65	14.08	0.07
Future-perspicuity	**3.94**	0.59	0.77	**4.14**	0.64	0.72	7.97	0.03
Future-planning	3.45	0.53	0.66	3.47	0.75	0.70	0.15	0.00
Self-esteem	**3.47**	0.50	0.78	**3.61**	0.74	0.80	3.78	0.01

*The bold fonts are significant difference.*

Rosenberg (1965) initially developed the Self-Esteem Scale; Wang et al. (1999) generated the modified Chinese version. The scale measures general self-evaluation on a single dimension and consists of 10 items, which are administered on a four-point scale, where one indicates “strongly agree” and four indicates “strongly disagree.” In this study, Cronbach’s α for the Chinese participants was 0.78, and Cronbach’s α for the American participants was 0.80.

### Procedure

Permission was obtained from the parents and teachers of the Chinese participants before conducting the survey in class. A graduate student was trained to supervise participants’ completion of the questionnaire. The American participants were students taking psychology classes, who participated in the survey online for course credit. All participants signed a consent form before the survey was conducted.

## Results

### Descriptive Statistics and ANOVAs

Descriptive statistics for the American and Chinese undergraduates are presented in Table 1. Multivariate analysis of variance (ANOVAs) showed that FTP dimensions and self-esteem differed between American and Chinese undergraduates (Wilk’s λ = 0.73, *F* (7, 792) = 41.64, *p* < 0.001, partial η^2^= 0.27). Univariate ANOVAs revealed that compared to Chinese undergraduates, American undergraduates were more negative [*F* (1, 798) = 35.94, *p* < 0.001, η^2^ = 0.08] and more confused [*F* (1, 798) = 20.14, *p* < 0.001, η^2^ = 0.04] about the future. American undergraduates were also more positive, perseverant and perspicuous about the future than Chinese undergraduates (*F*_all_ > 7.67, *p* < 0.01, η^2^_all_ > 0.02). American undergraduates also have higher self-esteem than Chinese [*F* (1, 798) = 3.78, *p* < 0.01, η^2^ = 0.01]. The difference between American and Chinese undergraduates was not significant for future-planning [*F* (1, 798) = 0.04, *p* > 0.05].

### Correlations Analysis

Pearson product-moment correlation analyses of the relationship between FTP and self-esteem (see Table 2) showed that among Chinese undergraduates, the future-positive (*r* = 0.45, *p* < 0.001), future-perseverant (*r* = 0.40, *p* < 0.001), future-perspicuity (*r* = 0.30, *p* < 0.05), and future-planning (*r* = 0.31, *p* < 0.001) subscales were positively correlated with self-esteem; whereas future-negative (*r* = -0.58, *p* < 0.001) and future-confusion (*r* = -0.48, *p* < 0.001) subscales were negatively correlated with self-esteem. For American undergraduates, the future-positive (*r* = 0.55, *p* < 0.001), future-perseverant (*r* = 0.41, *p* < 0.001), future-perspicuity (*r* = 0.51, *p* < 0.05), and future-planning (*r* = 0.19, *p* < 0.001) subscales were positively correlated with self-esteem; whereas the future-negative (*r* = -0.63, *p* < 0.001) and future-confusion (*r* = -0.35, *p* < 0.001) subscales were negatively correlated with self-esteem. This indicates that the correlations between components of FTP and self-esteem were similar in both cultures.

**Table 2:** Correlations between future time perspective and self-esteem.

	1	2	3	4	5	6	7
1. Future-negative	–	-0.64^***^	0.65^***^	-0.36^**^	-0.55^***^	-0.23^*^	-0.63^***^
2. Future-positive	-0.35^***^	–	-0.50^**^	0.48^***^	0.72^***^	0.24^**^	0.55^***^
3. Future-confusion	0.61^**^	-0.31^***^	–	-0.30^***^	-0.42^***^	-0.27^*^	-0.35^***^
4. Future-perseverant	-0.33^***^	0.40^***^	-0.22^***^	–	0.55^***^	0.42^***^	0.41^***^
5. Future-perspicuity	-0.42^***^	0.39^***^	-0.28^***^	0.43^***^	–	0.23^***^	0.51^***^
6. Future-planning	-0.31^***^	0.35^***^	-0.28^***^	0.47^***^	0.38^***^	–	0.19^***^
7. Self-esteem	-0.58^***^	0.45^***^	-0.48^***^	0.40^***^	0.30^***^	0.31^***^	–

*Correlations for Chinese undergraduates are shown below the diagonal line and those for American undergraduates above the diagonal line. ^∗^p < 0.05. ^∗∗^p < 0.01. ^∗∗∗^p < 0.001.*

### Hierarchical Regression Analysis of Future Time Perspective on Self-Esteem

Hierarchical regression analyses of the relationship between FTP and self-esteem were performed separately for American and Chinese undergraduates (see Table 3). Among Chinese undergraduates, after controlling for age, gender, and family economic status [model *F* (9, 450) = 53.65, *p* < 0.001, Δ*R* = 0.41], the significant predictors of self-esteem were family economic status (*β* = 0.08, *t* = 2.13, *p* < 0.05), the future-negative subscale (*β* = -0.35, *t* = -7.70, *p* < 0.001), the future-positive subscale (*β* = 0.22, *t* = 5.37, *p* < 0.001), the future-confusion subscale (*β* = -0.16, *t* = -3.53, *p* < 0.001), and the future-perseverant subscale (*β* = 0.16, *t* = 4.06, *p* < 0.001). Among American undergraduates, after controlling for age, gender and family economic status [model *F* (9, 330) = 74.92, *p* < 0.001, *ΔR* = 0.43], the significant predictors of self-esteem were the future-negative subscale (*β* = -0.53, *t* = -9.27, *p* < 0.001), the future-positive subscale (*β* = 0.15, *t* = 3.69, *p* < 0.05), the future-confusion subscale (*β* = -0.16, *t* = -3.18, *p* < 0.01), the future-perseverant subscale (*β* = 0.17, *t* = 3.75, *p* < 0.001), and (marginally) the future-perspicuity subscale (*β* = 0.11, *t* = 1.60, *p* < 0.1).

**Table 3:** Hierarchical regression analysis of future time perspective on self-esteem.

	China		America	
	Model 1	Model 2	Model 1	Model 2
**Step1**				
Gender	-0.10	-0.03	-0.03	-0.01
Age	-0.01	0.02	-0.09	-0.01
Family economic status	0.18^***^	0.08^*^	0.16^*^	0.05
**Step2**				
Future-negative		**-0.35^∗∗∗^**		**-0.53^∗∗∗^**
Future-positive		**0.22^∗∗∗^**		**0.15^∗∗∗^**
Future-confusion		**-0.16^∗∗∗^**		**-0.16^∗∗^**
Future-perseverant		**0.16^∗∗∗^**		**0.17^∗∗∗^**
Future-perspicuity		0.06		**0.11^†^**
Future-planning		0.01		0.01
*R*^2^	0.04	0.45	0.04	0.47
*F*	6.64	53.65^***^	2.60 ^†^	74.92^***^
*df*	(3, 456)	(9, 450)	(3, 330)	(9, 330)
Δ*R*		0.41		0.43

*Marginal significance ^†^p < 0.1. The bold fonts are significant predictive effect. ^∗^p < 0.05, ^∗∗^p < 0.01, and ^∗∗∗^p < 0.001.*

## Discussion

This study revealed that scores on the different dimensions of FTP varied by cultural background. Specifically, American undergraduates were more negative and confused about the future, but also more positive, perseverant, and perspicuous about the future than Chinese undergraduates. These findings are somewhat similar to one study (Gao, 2016), which showed that Chinese individuals regarded positive aspects of one’s future (expectations) as more important than Americans, whereas Americans regarded negative aspects of one’s future (fear) as more important than Chinese. Below, we elaborate on possible reasons for these differences.

First, since the financial crisis of 2008, the economic outlook in America has not been favorable (in 2018, the growth rate of the American GDP was only 2.9%; U.S. Department of Commerce, 2019). Hence, American undergraduates might have adopted a pessimistic attitude toward the future when considering their career and economic prospects. In contrast, China is a developing nation, with good developmental trends in recent years (in 2018, the growth rate of the Chinese GDP was 6.6% and the registered urban unemployment rate was 3.8%; National Bureau of Statistics of China, 2019). Therefore, Chinese undergraduates’ lower levels of pessimism about the future might be related to national socio-economic factors (Seginer and Schlesinger, 1998; So et al., 2016). However, American undergraduates also believe that the future can be predicted based on previous national trends in economic development, which could explain why they demonstrated more optimism about the future.

Second, American culture emphasizes individualism (Earley, 1989; Forbes et al., 2009; Jiang et al., 2016), which focuses on the autonomy of individuals. Undergraduates need to face the issue of employment after graduation. Thus, in addition to the aforementioned economic problems, they will also have to strive for their future in relative isolation, which might cause them to feel that the future is uncertain (Lee, 2012). However, members of individualistic cultures also tend to believe that their own efforts can make a difference. In contrast, Chinese culture emphasizes collectivism (Earley, 1989; Forbes et al., 2009; Jiang et al., 2016), which focuses on social harmony and dependency. Thus, Chinese undergraduates think about their future not only from their own perspective, but also from the perspective of important people around them (e.g., their parents, teachers, relatives, and friends) (Zhang et al., 2015). The various elements of “me” are interwoven, thus presenting a more complex view of the future.

Third, Eastern cultures often emphasize Confucianism, which advocates that “happiness lies in contentment.” Thus, past and present contentment is used as a basis for increasing the frequency and expectations of future thinking (Fingerman and Perlmutter, 1995). Our study discovered that American undergraduates exhibited greater perseverance regarding the future, and persistence implies the ability to resist current temptations. Bembenutty and Karabenick (2004) proposed that individuals delay gratification based on two aspects of information: the value of the delayed option and motivation to achieve the final goal. Individualism stresses individual freedom and realization of self-worth, which are complementary to the experience of achieving one’s ultimate goals. In particular, the one-child policy in China has created a large number of one-child families, and a superior childhood growth environment can lead to greater persistence. Moreover, influenced by the Confucian culture, Chinese students are more accustomed to the *Zhongyong* (The Doctrine of the Mean). Therefore, they show a tendency to be “not pleased by external gains, not saddened by personal losses.” In addition, the development of self-esteem also reflects the development of self-consciousness, which has also been demonstrated in our study, as American undergraduates scored higher in self-esteem than Chinese undergraduates (Farruggia et al., 2004; Cai et al., 2007). Accordingly, Americans undergraduates were more persistent about the future than Chinese undergraduates.

This study also showed that the relationship between FTP and self-esteem was consistent within two cultural environments. In both China and America, age and gender were not significant predictors of self-esteem. Future-negative subscale scores negatively predicted self-esteem, while future-positive subscale scores positively predicted self-esteem. This indicates that affect with respect to the future predicted self-esteem. Studies have shown that emotions are correlated with self-esteem (Zhang et al., 2016); individuals with past experiences of positive emotions tend toward high self-esteem, whereas those with past experiences of negative emotions tend toward low self-esteem (Lyu and Huang, 2008). Similar to the present study, Kang et al. (2003) found that the impact of emotional status on self-esteem was consistent across cultures, which suggests that an individual’s positive self-perception is affected to a certain extent by temperament rooted in biological underpinnings (Schimmack et al., 2002). Future-perseverance subscale scores were also a significant predictor of self-esteem. Future-perseverance primarily involves behavioral persistence and manifests as an individual’s ability to delay gratification (Lyu and Huang, 2016). When individuals perform a comprehensive evaluation of their surrounding environment and engage in long-term planning, the process of choosing between delayed and immediate gratification cannot be separated from self-evaluation and regulation (Bembenutty and Karabenick, 2004). Selecting delayed gratification implies self-regulation and positive self-evaluation.

It should be noted that future confusion negatively predicted self-esteem, whereas future-perspicuity positively predicted self-esteem, among American undergraduates, which reflects different effects of different types of awareness about one’s future. From the perspective of the self, future-confusion stems from present uncertainty. Given that most of the undergraduate participants in the current study had just transitioned from high school into a university setting, they were faced with uncertainty in an unfamiliar environment, thereby engendering an unstable cognitive evaluation of the self (Ross, 1995). However, independence is fostered among Americans from early childhood; hence, American undergraduates may been better able to cognitively cope with changes in their surrounding environment, and being certain about oneself and one’s surroundings is positively correlated with self-evaluations (Orr and Moscovitch, 2015). Therefore, Chinese undergraduates possibly tended to change their self-concept to adapt to their environment, whereas American undergraduates possibly tended to change their environment to adapt to their self-concept (Jiang et al., 2016).

Furthermore, family economic status was positively predictive of self-esteem, whereby better economic status was associated with higher levels of self-esteem. This finding is consistent with previous studies. For example, studies have shown that self-esteem and socio-economic status (including family economic status) are positively correlated (Zhang and Postiglione, 2001; Twenge and Campbell, 2002). Undergraduates with better family economic status will experience greater social support and encounter different social challenges, thus continuously improving their self-evaluation during this process. As China is still in the developmental stage, there is still a significant difference in family economic status caused by the wealth gap. A meta-analysis by Twenge and Campbell (2002) showed that members of Asian cultures believed that socio-economic status was particularly important to self-esteem, which reflects the self-protection mechanism that exists in those with collectivistic backgrounds.

It should be noted that future-planning subscale scores did not significantly differ according to cultural background. This indicates that regardless of the environment, future-planning is indispensable. When undergraduates break away from the constraints of their families, they will require clear understanding so as to plan for the future and better adapt to society. This is a manifestation of universal psychological adaptability in humans (Londono and McMillan, 2015).

Of course, there were a few limitations in this study. First, the sample’s representativeness was limited, as only three cities (Chongqing in China and Chicago, Illinois and Athens, Ohio in America) were selected for this study. The sample size should also be increased in future studies. Furthermore, sample homogeneity within the two cultures could not be ensured, with relatively large influences from external factors. Thus, caution is needed when generalizing the research conclusions. Future studies should include more countries (e.g., Japan, Korea, Britain, France) to increase generalizability. Second, FTP and self-esteem are two relatively stable traits, and there were no differences across cultures in their relationship to one another, possibly because the scale wording was not culture-specific. It is necessary to examine implicit as well as implicit measures of these constructs (e.g., future fluency task, implicit self-esteem) in further research. Finally, the FTP scale may have been limited by potential differences in comprehension of language across cultures. That is, errors might have occurred during the translation process, and hence, better measurement tools should be developed in subsequent studies.

In conclusion, compared to Chinese students, American students were more negative and more confused about the future, but were also more positive, persistent and perspicuous about the future than Chinese students. In both the American and Chinese samples, the future-negative, future-positive, future-confusion, and future-perseverant subscales of FTP significantly predicted self-esteem.

## Ethics Statement

This study was approved by the ethics committee of Faculty of Psychology at Southwest University. The participants signed an informed consent form stating the aim of the study and explaining that they could withdraw from the study, and the data would be anonymous.

## Author Contributions

HL designed the study idea and research framework. GD contributed to the data analysis and writing. KR contributed to the data collection and manuscript modification.

## Conflict of Interest Statement

The authors declare that the research was conducted in the absence of any commercial or financial relationships that could be construed as a potential conflict of interest.
